# *CRY2* Is Associated with Depression

**DOI:** 10.1371/journal.pone.0009407

**Published:** 2010-02-24

**Authors:** Catharina Lavebratt, Louise K. Sjöholm, Pia Soronen, Tiina Paunio, Marquis P. Vawter, William E. Bunney, Rolf Adolfsson, Yvonne Forsell, Joseph C. Wu, John R. Kelsoe, Timo Partonen, Martin Schalling

**Affiliations:** 1 Neurogenetics Unit, Department of Molecular Medicine and Surgery, Karolinska Institutet at Karolinska University Hospital Solna, Stockholm, Sweden; 2 Public Health Genomics Unit, Department of Chronic Disease Prevention, National Institute for Health and Welfare, Helsinki, Finland; 3 Functional Genomics Laboratory, Department of Psychiatry and Human Behavior, University of California Irvine School of Medicine, Irvine, California, United States of America; 4 Division of Psychiatry, Department of Clinical Sciences, University of Umeå, Umeå, Sweden; 5 Department of Public Health Science, Karolinska Institutet at Karolinska University Hospital Solna, Stockholm, Sweden; 6 Department of Psychiatry, University of California San Diego School of Medicine, La Jolla, California, United States of America; 7 Mood, Depression and Suicidal Behaviour Unit, Department of Mental Health and Substance Abuse Services, National Institute for Health and Welfare, Helsinki, Finland; University of Wuerzburg, Germany

## Abstract

**Background:**

Abnormalities in the circadian clockwork often characterize patients with major depressive and bipolar disorders. Circadian clock genes are targets of interest in these patients. *CRY2* is a circadian gene that participates in regulation of the evening oscillator. This is of interest in mood disorders where a lack of switch from evening to morning oscillators has been postulated.

**Principal Findings:**

We observed a marked diurnal variation in human *CRY2* mRNA levels from peripheral blood mononuclear cells and a significant up-regulation (P = 0.020) following one-night total sleep deprivation, a known antidepressant. In depressed bipolar patients, levels of *CRY2* mRNA were decreased (P = 0.029) and a complete lack of increase was observed following sleep deprivation. To investigate a possible genetic contribution, we undertook SNP genotyping of the *CRY2* gene in two independent population-based samples from Sweden (118 cases and 1011 controls) and Finland (86 cases and 1096 controls). The *CRY2* gene was significantly associated with winter depression in both samples (haplotype analysis in Swedish and Finnish samples: OR = 1.8, P = 0.0059 and OR = 1.8, P = 0.00044, respectively).

**Conclusions:**

We propose that a *CRY2* locus is associated with vulnerability for depression, and that mechanisms of action involve dysregulation of *CRY2* expression.

## Introduction

Rhythms that approximate the 24-hour day-night cycle or light-dark transitions are called circadian. Abnormalities of the circadian pacemaker system is often seen in mood disorders (i.e. major depressive and bipolar disorders) as evidenced with sleep stage abnormalities and the clinical efficacy of sleep deprivation [1] as well as with the therapeutic mechanisms of lithium [2] that is prescribed primarily for bipolar disorder. Approximately one tenth of all mood disorders follow a seasonal pattern and area hence categorized as seasonal affective disorder (SAD) [3]. Season-bound mood episodes may occur in both depressive and bipolar disorders and emerge in any season, but the most common type is winter depression, a condition in which major depressive episodes routinely occur in the wintertime and remit the following summer [4]. In one study as much as 93% of the winter depression cases had a diagnosis of bipolar disorder [5] although other studies tend to show a greater proportion of unipolar recurrent major depression among winter depression cases [4]. Depressive episodes in winter depression are highly recurrent and appear to be clearly endogenous as there is no psychosocial factor that would account for their onset. For bipolar type 1 disorder, the heritability estimate is very high [6]. Therefore, winter depression, whether part of major depressive disorder or bipolar disorder, as well as bipolar type 1 disorder, with or without a seasonal pattern, provide excellent models for studying the molecular mechanisms of mood disorder [7], [8].

Advances and delays in phase (the location within a cycle at a particular time) and reduced amplitudes (intensities of the oscillations) have been reported in adult patients [9]–[11], while children with winter depression have circadian rhythms that are accurate in phase but low in amplitude [12]. This agrees with the hypothesis that not only phase shifts but also amplitude attenuations [13] contribute to the pathogenesis, and suggests that there are circadian clockwork abnormalities having relevance to the daily reset and synchronization. This is further supported by findings of the seasonal changes in sensitivity to light exposure in winter depression, these patients having supersensitivity to light in terms of melatonin suppression during winter [14], abnormal melatonin levels in patients with seasonal or non-seasonal depressive disorder [15], and abnormalities in circadian alignments in patients with bipolar disorder [16]. Therefore, the internal misalignment (i.e. the sleep-wake cycle is no longer in phase with the circadian rhythms) may account for the pathogenesis of mood disorders in general. Light exposures and sleep manipulations in patients with mood disorder are tools for exploration and elucidation of the mechanisms driving the circadian and seasonal clockworks.

CRY proteins [17] differ from many transcription factors that take part in the circadian clockwork, since they have no PAS domains. This suggests that they are unique in the core of the circadian clockwork where they act [18]. Both CRY1 and CRY2 operate in the retina and non-visual light detection pathways in a manner that is important for the internal alignment [19]–[24]. Of these two, CRY2 is highly expressed in the brain in particular and has a dose-dependent inhibitory effect on the activated ARNTL, whereas both CRY2 and CRY1 repress all four combinations of the ARNTL (ARNTL2) – CLOCK (NPAS2) protein heterodimers that act as transcriptional activators in the core of the circadian clockwork [25]. These basic findings give a rationale for the *CRY2* gene as a target of high interest and relevance in our study.

Here, we assessed *CRY2* gene expression in eight healthy volunteers for 48 hours and in 13 patients with bipolar disorder before and after a one-night sleep deprivation, and we report that *CRY2* mRNA levels are reduced and unresponsive to sleep deprivation in depressed patients with bipolar disorder. To determine whether *CRY2* genetic variation is associated with depression, we analyzed circadian clock gene variants in two separate population-based samples, a Swedish sample and a Finnish sample, and report that the *CRY2* gene is associated with winter depression.

## Results

### *CRY2* Expression Analysis

**Figure 1:**
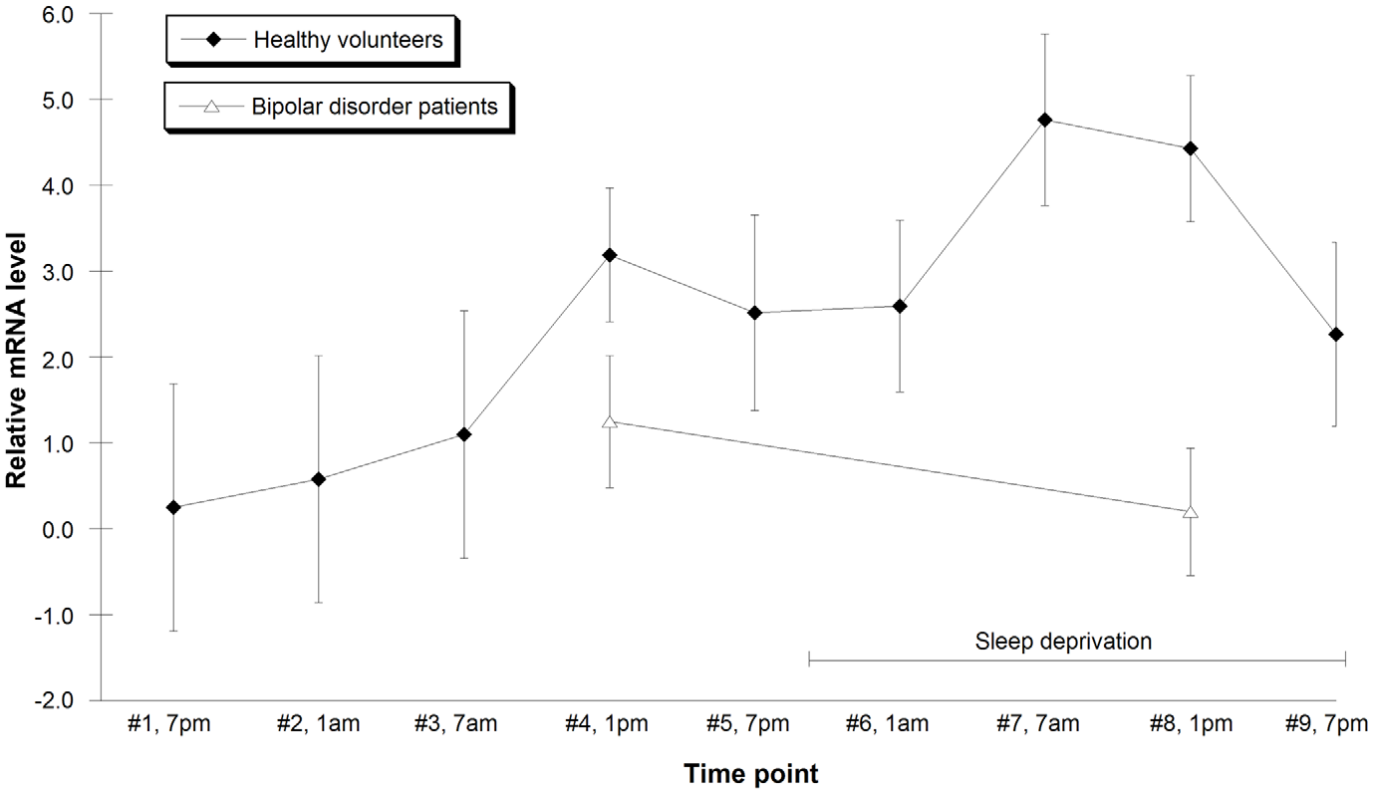
*CRY2* gene expression in healthy volunteers and bipolar disorder patients. *CRY2* mRNA expression was not changed by sleep deprivation in bipolar disorder patients (triangle) and was significantly decreased compared to healthy volunteers (square) during sleep deprivation. Blood draw 1 (x-axis) was at 7 p.m., then there was a blood draw every 6 hours. Sleep deprivation was at time points #6, #7, #8 and #9. Bars indicate mean and error bars indicate SEM.

*CRY2* mRNA levels displayed marked diurnal variation in healthy controls (n = 8). In addition, total sleep deprivation induced a 2.0-fold increase in *CRY2* mRNA levels (P = 0.020) in the controls (Figure 1). The control gene *GAPDH* mRNA levels did not show diurnal variation and were not changed by the effect of sleep deprivation (P = 0.66).

In samples from patients in a depressive state of bipolar disorder (n = 13), sleep deprivation did not induce any increase in *CRY2* mRNA levels. *CRY2* mRNA expression was significantly decreased in the samples from patients as compared with the samples from healthy controls during sleep deprivation (P = 0.041 for the diagnosis and sleep deprivation interaction using ANOVA; P = 0.029 for the difference between patients and controls during sleep deprivation in post hoc test). *GAPDH* showed similar mRNA levels in the samples from controls to those in the bipolar disorder samples (P = 0.12).

### Genetic Association Analysis

**Figure 2:**
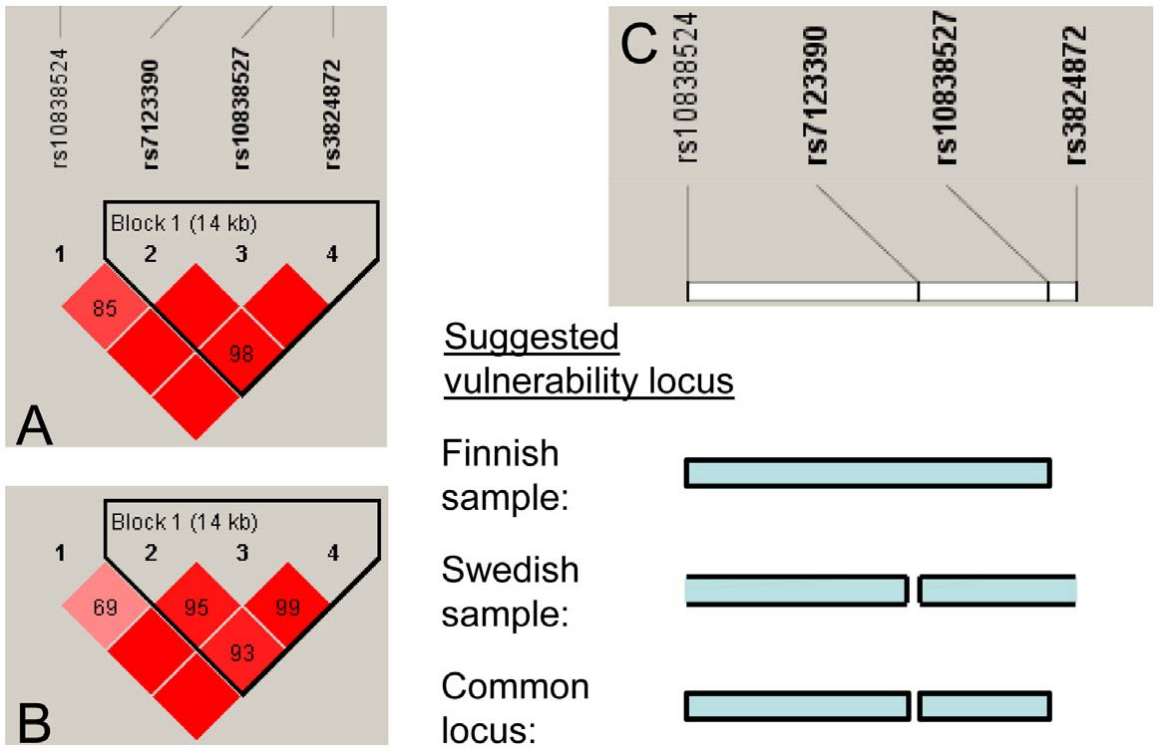
The suggested vulnerability locus in *CRY2*. LD structure of the SNPs analyzed in A) the Swedish sample, and B) the Finnish sample. C) Scheme of the suggested location of the functional variant in the Finnish sample, in the Swedish sample, and the common locus relevant under the assumption of the same functional variant in the two samples. An open end of locus box (seen for the Swedish sample) indicates that this is not the locus border. The numbers in the squares of A and B) represent the pair-wise D' value, empty squares stand for D' = 1. Pink-red color indicates a pair-wise LOD ≥2 with redness proportional to D'. Haplotype blocks are formed if 95% of comparisons are “strong LD”, i.e. the 95% CI of D' is within [0.7–0.98].

Analyses were performed on two samples of clinical cases with a diagnosis of winter depression (DSM-IV) and healthy screened controls (Swedish sample: 118 cases and 1011 controls, Finnish sample: 86 cases and 1096 controls). *CRY2* allele frequency was significantly associated with winter depression in the Swedish sample, as shown with three SNPs (rs10838524 risk allele A, rs10838527 risk allele G, and rs3824872 risk allele A) having OR = 1.6, OR = 2.1 and OR = 1.8 (P = 0.0017, P = 0.00074 and 0.00070, respectively). The association to rs10838524 was confirmed in the Finnish sample (OR = 1.7, P = 0.0020) (Table 1) and indicated that homozygosity of the minor allele (overall minor allele frequency of about 0.5) was increased in the cases in both population samples (Table 2). The associated SNPs (rs10838527 and rs3824872) were present in LD block 1 (rs7123390, rs10838527, rs3824872) in both samples (Figures 2A and 2B).

**Table 1:** SNP allele frequency association analysis.

SNP	Location	Alleles	MAF: A/U	OR [95% CI] a	*P* value a	OR [95% CI] b	Empirical *P* b,c
Swedish sample							
rs10838524	Intron 1	A*/G	0.54/0.45	1.65 [1.21–2.25]	0.0017	1.41 [1.07–1.86]	0.013
rs7123390	Intron 7 (50 bp from exon7)	A/G*	0.28/0.28	0.97 [0.69–1.37]	0.88	1.00 [0.73–1.40]	0.96
rs10838527	Exon 12 in 3′ UTR	G/A*	0.13/0.09	2.11 [1.37–3.26]	0.00074	1.73 [1.15–2.61]	0.010
rs3824872	Downstream 3′ UTR	A/C*	0.27/0.20	1.76 [1.27–2.43]	0.00070	1.49 [1.09–2.04]	0.014
Finnish sample							
rs10838524	Intron 1	G/A*	0.60/0.47	1.69 [1.21–2.36]	0.0020	1.72 [1.24–2.39]	0.0021
rs7123390	Intron 7 (50 bp from exon7)	A/G*	0.19/0.29	0.60 [0.40–0.91]	0.015	0.59 [0.40–0.87]	0.016
rs10838527	Exon 12 in 3′ UTR	G/A*	0.089/0.13	0.68 [0.39–1.19]	0.18	0.68 [0.39–1.19]	0.18
rs3824872	Downstream 3′ UTR	A/C*	0.20/0.26	0.70 [0.47–1.05]	0.089	0.70 [0.47–1.05]	0.089

Alleles, minor allele first.

OR, proportion of minor versus major allele among affected (A)/proportion of minor versus major allele among non-affected (U).

a Logistic regression with gender as covariate. *P*<0.05 are shown.

b No covariate.

c Point-wise *P-*value from 10,000 permutations with no covariate (EMP1).

* Ancestral allele in CEU population data (CEPH (Utah residents with ancestry from northern and western Europe)) from www.hapmap.org.

**Table 2:** Genotype association analysis of the SNPs with suggestive allele frequency association.

SNP	Cases aa/ab/bb (%)	Cases *n*	Controls aa/ab/bb (%)	Controls *n*	Cochran-Armitage trend *P* value	Minor allele dominant *P*	Minor allele recessive *P*
Swedish sample							
rs10838524	30/48/22	113	20/51/29	906	0.014	0.14	0.011
rs10838527	3.5/20/76	113	0.52/16/84	951	0.0085	0.048	0.010
rs3824872	13/28/58	113	4.2/32/64	927	0.014	0.27	0.000041
Finnish sample							
rs10838524	38/43/18	76	22/49/29	1 039	0.0015	0.047	0.0014
rs7123390	3.8/32/64	78	7.9/42/52	1 066	0.014	0.019	0.27

Allele ‘a’ is the minor allele.

Note that the identity of the rs10838524 minor allele differs between the Swedish and Finnish samples.

Four haplotypes were identified and a significant difference in distribution of haplotypes between the cases and controls was found for the Swedish sample (χ^2^ = 8.7, P = 0.034) and the Finnish sample (χ^2^ = 14.6, P = 0.0022; see Supplemental data Table S1). A haplotype of the risk alleles GGA was more frequent in the Swedish cases compared with controls (OR = 1.7, P = 0.012) and even more so when adding the rs10838524 risk allele to the haplotype to be AGGA (OR = 1.8, P = 0.0059; Table 3). The block 1 (rs7123390, rs10838527, rs3824872) haplotype GAC which in the Swedish sample has the protective alleles from rs10838527 and rs3824872 was a risk haplotype in the Finnish sample (OR = 1.8, P = 0.00010), and similarly so when adding the rs10838524 allele G to the haplotype to be GGAC (OR = 1.8, P = 0.00044; Table 3). The Finnish data suggest a vulnerability locus increasing the risk for winter depression upstream rs10838527. This was further supported by the fact that changing only the rs7123390 allele in the Finnish risk haplotype (GAC) turned it into a protective haplotype (AAC) in the Finnish sample (OR = 0.61, P = 0.012), and the fact that the AGAC was non-existent of in the samples. Considering only the Swedish data the vulnerability locus could reside on either side of, but not exactly at rs7123390. If assuming the same functional variation (although probably opposite risk alleles in Swedes and Finns), the Swedish and the Finnish samples together hence suggest the vulnerability locus to reside in-between rs7123390 and rs10838527 or upstream rs7123390 (Figure 2C).

**Table 3:** Haplotype association analysis.

SNPs	Haplotype	Frequency cases	Frequency controls	OR [95% CI]	*P* value
Swedish sample					
rs7123390-rs10838527-rs3824872	GGA	0.14	0.090	1.68 [1.13–2.53]	0.012
rs7123390-rs10838527-rs3824872	GAC	0.44	0.52	0.74 [0.56–0.97]	0.032
rs10838524-rs7123390-rs10838527-rs3824872	AGGA	0.14	0.082	1.82 [1.17–2.63]	0.0059
rs10838524-rs7123390-rs10838527-rs3824872	GGAC	0.44	0.51	0.75 [0.57–0.98]	0.048
Finnish sample					
rs7123390-rs10838527-rs3824872	AAC	0.21	0.29	0.61 [0.42–0.90]	0.012
rs7123390-rs10838527-rs3824872	GAC	0.59	0.45	1.84 [1.35–2.55]	0.00010
rs10838524-rs7123390-rs10838527-rs3824872	AAAC	0.20	0.27	0.67 [0.45–0.98]	0.046
rs10838524-rs7123390-rs10838527-rs3824872	GGAC	0.59	0.45	1.76 [1.31–2.47]	0.00044

Odds ratio (OR): the ratio specific haplotype versus all other haplotypes among the cases, relative to the ratio specific haplotype versus all other haplotypes among the controls.

## Discussion

Our key findings herein are that *CRY2* gene variation and expression levels are associated with depression. *CRY2* mRNA levels are lowered in blood mononuclear cells from depressed patients with bipolar disorder after total sleep deprivation in comparison to healthy controls, and *CRY2* gene variation was associated with winter depression in both Swedish and Finnish patients. Differences in *CRY2* risk haplotypes were observed between the Swedes and Finns. Overall there are genetic differences between these populations [26]. The fact that Swedes and Finns have different histories in terms of going through genetic bottle-necks that could have affected selection in operation at the *CRY2* locus with regard to depression vulnerability, would possibly explain the differences in *CRY2* risk haplotypes seen between the two samples, and explain a putative allelic heterogeneity (different vulnerability alleles in Swedes and Finns) of the functional *CRY2* polymorphism. Assuming the same functional variant in the Swedish and the Finnish samples, the two samples would assist in narrowing the interval of that functional variant (Fig. 2C). The risk haplotypes spanned from *CRY2* intron 1 to 3′UTR, and pointed at a location of a potential function variation somewhere upstream exon 12 in 3′UTR (rs10838527) from the Finnish data, with a distance from the intron 1 marker rs10838524 to rs10838527 corresponding to ∼33 kb (NCBI build 130). The vulnerability locus from the Swedish data overlapped the Finnish locus, but for at the intron 7 marker rs7123390, and extended downstream rs10838527.

Human *CRY2* mRNA levels have been demonstrated to undergo circadian oscillation in fibroblasts [27] and in hematopoietic stem cells [28]. Our results herein add on these findings and demonstrate the effect of sleep deprivation on the circadian oscillations of *CRY2* mRNA in human peripheral blood mononuclear cells. Our data indicate that depression in bipolar disorder is related to lowered levels of *CRY2* mRNA. Sleep deprivation led to *CRY2* mRNA increase in controls whereas depressed bipolar individuals were non-responsive.

CRY2 has been purified from human cells and its properties have been described [29], but the understanding of *CRY2* regulation and CRY2 functions still need elucidation. Earlier studies have demonstrated that *CRY2* transcription is driven by the canonical circadian circuit but through a unique mechanism of action not yet characterized [30]–[32]. *CRY2* is unique among the canonical genes of the circadian clock, since experimental findings of its effects on the circadian clockwork violate the predictions from a theory on the roles of PER1, PER2, CRY1 and CRY2 [33]–[36]. Deletion of *Cry2* gene lengthened the circadian period by approximately 48 min (from 23.7 to 24.5 hours) [37] and in fact strengthened circadian amplitudes and restored the lost rhythms in *Per2* mutant mice [38], [39]. These findings fit in the view that activation of *Per1* and *Per2* genes occurs in the morning, whereas activation of *Cry1* and *Cry2* genes occurs in the evening [40]. Abnormalities in *CRY2* regulation would therefore leave the morning oscillator intact, which agrees with findings in patients with winter depression [41] and those with bipolar disorder [42]. However, disruption of *CRY2* regulation may compromise the evening oscillator and the subsequent proper oscillator reactions to light-dark transitions [43]. *Cry2* null mutants are less decelerated, or are more accelerated, by light exposure than *Cry1* null mutants [36]. The former phenotype does match with that seen in winter depression [44] and, to some extent, in bipolar type 1 disorder [45].

The action of melatonin on *Cry1/Cry2* expression is hypothesized to form the basis of refractory reactions to stimulation with light [40], and the subsequent subsensitive or supersensitive responses to light exposure. Phase control through the melatonin-guided interval of *Per1/Per2* to *Cry1/Cry2* expression peaks may have relevance to mood disorders, as the internal alignment and the melatonin signal (amplitude and phase) are abnormal in patients with bipolar disorder and those with winter depression in particular [15], [16], [46]. Our findings now suggest that a dawn component [47], [48] and a dusk component (herein), i.e. *PER2* and *CRY2* variants respectively, are affected in winter depression. Such influence may well contribute to phase angle differences [49] and entrainment errors [11] that have been found in patients with winter depression.

To sum up, the phenotype of the *Cry2* knockout mice together with mechanistic data are in line with the finding in this report suggesting that *CRY2* has a role in winter depression.

Our current findings add to earlier gene expression studies in blood that have reported a number of potential peripheral biomarkers for bipolar disorder [50], [51], and earlier gene expression findings in brain indicating that clock genes are implicated in a model of bipolar disorder, the first report being [52], followed by [53] for *CRY2*. Circadian genes with a polymorphism previously reported to be associated to human bipolar disorder include *CLOCK* [54], and *NR1D1*, *ARNTL* and *PER3* [55]–[57]. Moreover, *ARNTL*, *RORA*, *RORB* and *RXRG* were associated with bipolar disorder in a meta-analysis integrating data from genome-wide association studies and human and animal model expression studies [51]. *PER2*, *NPAS2* and *ARNTL* genetic variation have been found associated with winter depression [47], [48], [58], and recent experimental data demonstrated these three proteins to regulate transcription of *MAOA* and to have subsequent influence on depressive behavior [59].

Other circadian genes were tested for association to winter depression in parallel with *CRY2*. The *CRY2* finding reached significance even after correction for multiple testing considering other circadian gene SNPs tested for association to winter depression in parallel with *CRY2*. The corrected threshold for significance corresponded to a nominal P-value of 0.001 and was calculated applying a Bonferroni correction considering the partial LD between markers [60], [61]. As a novel finding we report here that CRY2 expression is reduced and non-responsive to sleep deprivation in depressed patients with bipolar disorder, suggesting that *CRY2* plays a role in bipolar disorder.

There are some limitations in our work. A limitation in the *CRY2* expression analysis was the few time points for the cases, since they do not allow analysis of the *CRY2* rhythm and possible phase shift among the cases. However, irrespectively of whether there was a phase shift or not among the cases, *CRY2* mRNA levels were significantly lower in cases compared to controls after sleep deprivation, which suggests that *CRY2* levels is a trait marker that is non-responsive to the antidepressant sleep deprivation. Though, we cannot exclude that the *CRY2* mRNA levels in the patients were influenced by medication. All the patients were on an SSRI together with a mood stabilizer. Lithium is known to affect the circadian clock through inhibition of GSK3 [2], and there are reports of SSRI influencing the circadian rhythms [62]. Since all controls and 10 of the patients were studied during the summer, the season of the examinations does not likely explain the *CRY2* mRNA level difference between patients and controls. A limitation in the genetic analysis was the number of patients. However, the cases and the controls in both the Swedish and the Finnish materials represented ethnically homogeneous populations, and the controls were extensively scored to exclude any mental illness, reducing bias due to the ethnic variation and dilution of genetic effects due to disorder heterogeneity among the controls.

In conclusion, we show data suggesting that variation in *CRY2* links to depression. *CRY2* mRNA expression levels were lowered in PBMCs from depressed patients with bipolar disorder. In two ethnically homogeneous population-based samples, *CRY2* SNPs were associated with winter depression. Although this study contains a replication of the genetic findings, it remains limited due to the lack of replication of the expression findings, thus warranting further studies.

## Methods

### *CRY2* Expression Study

Informed consent was obtained from each participant using an approved University of California Institutional Review Board (IRB) protocol.

Thirteen patients (10 men, 3 women, out of which 11 with European, one with Asian and one with African decent) with bipolar type 1 disorder according to DSM-IV criteria, aged 40.2 years on average (SD = 13.4, range = 18–57), were enrolled in a sleep deprivation study at University of California Irvine (UCI) and San Diego (UCSD). During the study, all the patients were depressed, with the score on the 21-item Hamilton Depression Rating Scale [63] being 17.3 (SD = 6.4) on average. Eight healthy volunteers (4 women, 4 men, all with European decent), aged 23.6 years on average (SD = 5.9, range = 19–34 years), served as controls. Both the patients and the controls were hospitalized at the sleep research center at University of California at Irvine Medical Center (UCIMC) for 48 hours and deprived of sleep for 21 hours after an overnight stay (Figure 2). The season for the study of the patients was summer (n = 10) and winter (n = 3), and all controls were studied in the summer. All the patients were on an SSRI and either lithium (n = 8), valproate (n = 1) or lamotrigine (n = 4). None of the controls was on medication.

For the controls, venous blood samples were drawn at 9 different times, beginning at 7 p.m. and every 6 hours thereafter. For the patients, the blood was drawn at 1 p.m. on the days before and after sleep deprivation, corresponding to the time points #4 and #8 for the control samples. The patient blood was drawn in standard Vacutainer tubes without additive (10 ml, Becton Dickinson, Franklin Lakes, NJ, USA). From each control the blood was collected into both a standard acid citrate dextrose (ACD) tube (Becton Dickinson, Franklin Lakes, NJ, USA) as well as a Tempus Blood RNA Tube (Applied Biosystems, Foster City, CA, USA) in the same blood draw, yielding the total of 25–35 ml of whole blood. To check for potential difference in RNA degradation between control and bipolar disorder samples, the level of the house-keeping transcript *GAPDH* (glyceraldehyde-3-phosphate dehydrogenase) was analyzed with qPCR (data in the results section). To further rule out possible bias in comparison between the patients and controls due to type of collection tube, we compared *CRY2* and *GAPDH* mRNA levels using qPCR between the Tempus tube and the ACD tube from the controls collected at the same blood draw. The *CRY2* and *GAPDH* mRNA levels were not different between tube types (data not shown).

### Peripheral Blood Mononuclear Cells (PBMC) Isolation and RNA Extraction

All samples from both the patients and the controls were prepared similarly. Within a few minutes in room temperature after blood draw, the whole blood samples were layered onto Ficoll (Amersham Biosciences, Piscataway, NJ, USA), and PBMC were separated by density gradient centrifugation at 2500 rpm at room temperature for 20 min. The resulting ‘buffy’ coat at the interface was added to 5 ml phosphate buffered saline (PBS) at pH of 7.4 (Invitrogen, Carlsbad, CA, USA) and cell counts were taken. Cells were centrifuged at 1000 rpm for 10 min at room temperature. The resulting pellets were resuspended in 1 ml Trizol and stored at minus 80°C. Total RNA was extracted using the standard Trizol isolation protocol (Invitrogen, Carlsbad, CA, USA). The RNA was resuspended in 50 µL DEPC water, cleaned by passing over silica-based mini-spin columns (Qiagen RNeasy PlusMini Kit, Valencia, CA, USA) and analyzed for quality and quantity on a 2100 Bioanalyzer (Agilent, Palo Alto, CA, USA) and concentration was adjusted to 1 µg/µl.

### Real Time Quantitative PCR (qPCR)

The total RNA (1 µg RNA) from both the patients and the controls were identically synthesized into complementary DNA (cDNA) using Oligo d(T)_16_ primer and TaqMan Reverse Transcription Reagents and (Applied Biosystems, Foster City, CA, USA) at 25°C for 10 min, 48°C for 30 min and 95°C for 5 min. The quantitative PCR (qPCR) was performed on an ABI 7900HT Sequence Detection System (Applied Biosystems, Foster City, CA, USA) with triplicates for each cDNA, using Robot Biomek3000 (Beckman Coulter, Fullerton, CA, USA), with all samples run in the same plates with same normalization procedures. The reaction was performed in 12.5 µl consisting of 6.25 µl 2xSYBR Green Master Mix (Applied Biosystems, Foster City, CA, USA); 2.5 pmol of each primer; 2 µl 1∶10 dilution of cDNA template (corresponding approximately to 4 ng RNA). The thermal cycling profiles were 50°C for 2 min (incubation), 95°C for 10 min (activation), 45 cycles at 95°C for 15 sec (denaturation) and 60°C for 1 min (annealing/extension), then by dissociation step as 95°C for 15 sec, 60°C for 15 sec, and 95°C for 15 sec. The *CRY2* primer sequences spanned exon 6–7 junction within target sequence (Affymetrix probeset 3329058, transcript ID 3329029) and were: forward 5′-CCTACCTGCGCTTTGGTTGT-3′, reverse 5′-TGCTGTTCCGCTTCACCTTT-3′. The primers were pre-tested using brain cDNA, genomic DNA, no temple control (NTC) and RT minus on the ABI 7900HT. The results ensured good amplification of cDNA, and that neither residual genomic DNA nor primer-dimer contributed to measurements.

### Genetic Association Study

The local ethic committees (National Public Health Institute, Karolinska Institutet, and University of Umeå) approved the study protocol, and all the participants signed an informed consent after the protocol had been fully explained.

Patients were recruited from outpatient services at which senior specialists in psychiatry, familiar with our research program, were working. All patients met the DSM-IV diagnostic criteria for major depressive disorder with the seasonal (winter) pattern [64]. The consensus diagnosis of two independent psychiatrists was required for inclusion for each patient. Patients were from Sweden (118 patients: 13.6% men, 86.4% women) and Finland (86 patients: 29.1% men, 70.9% women), both being of local origin.

Controls matched for ethnicity and nationality (1011 Swedes: 39.2% men, 60.8% women; and 1096 Finns: 29.0% men, 71.0% women) had no current or past psychotic or mood disorder as assessed with interviews or self-rated questionnaires and were representative of the Swedish and Finnish adult populations. These population-based studies have been described more in detail earlier [65]–[67]. The Swedish cases and controls lived in Västerbotten and Stockholm areas. The Swedish population has no strong internal genetic borders [24]. The Finnish cases lived in the capital area (Helsinki and its surroundings), with inhabitants from all the regions in Finland, and the controls were collected from the nation-wide health examination study.

### Single-Nucleotide Polymorphism (SNP) Selection and Genotyping

Haplotype-tagging SNPs covering the variation in *CRY2* were selected using the HapMap database [68], applying the cut-off values of 0.8 for r^2^ and of 0.1 for the minor allele frequency (MAF). The genotyping was performed using the SEQUENOM iPLEX Application with the MassARRAY System (Sequenom, Inc., San Diego, CA, USA). To control for quality, 2.5% of the samples were genotyped in duplicates. The genotypes of these replicas were all in agreement.

The four *CRY2* SNPs genotyped fulfilled the criteria for SNP association test including a success rate of at least 90% of the reactions, and the Hardy-Weinberg equilibrium (HWE) with P>0.05 among the controls. The DNA samples from 10 Swedish cases and 53 controls, and 5 Finnish cases and 24 controls were excluded from analysis, since genotyping of them failed in more than 20% of a separate set of 111 SNP assays.

### Statistical Analyses

For the RNA analysis, the cycle threshold (Ct) was determined approximately in the middle of exponential phase of the amplification, and generally default condition on the software SDS 2.3 was used. The average Ct value was accepted if the CV was lower than 2% and SD was lower than 0.39. The Ct values for *CRY2* and the house-keeping gene *GAPDH* were normalized using the housekeeping genes *C1ORF82* and *TFG*. The selection of *C1ORF82* and *TFG* and the normalization method was based on the GENORM [69]. Within each of the case and the control groups, correlation for *CRY2* and *GAPDH* expression levels to age was tested and showed no significant correlation. Among controls, the *CRY2* and *GAPDH* expression levels were compared between pre-sleep deprivation time points (pooled time points from #1 to #4) and sleep deprived time points (pooled time points from #6 to #9), where the individual was used as a factor in a mixed model ANOVA.

*CRY2* and *GAPDH* expression levels comparison between bipolar patients (time point 1 p.m.) and controls (pooled time points from #1 to #4, and from #6 to #9) was done using ANOVA with the covariates gender, age and sleep deprivation (yes/no). Significant effect in ANOVA (p<0.05) was followed-up with post hoc test.

The SNPs were analyzed for allele frequency differences between the cases and controls in each population sample using logistic regression. Gender was used as a covariate, since women were overrepresented among Swedish cases compared with controls. To obtain empirical significance values, permutation tests with up to 10,000 permutations were performed. There was a similarity between the gender corrected effect size and the effect size when no gender was corrected for, especially in the Finnish sample (Table 1). Hence, the gender was not generally controlled for in the subsequent analyses. However, suggestive genotypic association findings in the Swedish sample were verified using logistic regression with the gender as a covariate, and displayed a very minor gender effect. The power was ≥0.8 to detect allele frequency differences at odds ratio (OR) of ≥1.8 in the Swedish sample and at OR of ≥2.0 in the Finnish sample when the MAF was of 0.1 to 0.3 and the non-corrected P was of <0.05 (http://pngu.mgh.harvard.edu/~purcell/gpc/cc2.html). Those SNPs whose allele frequency difference was indicative (P<0.05) were analyzed for genotype association. Calculations were performed using the PLINK program, version 1.04 [70].

The linkage disequilibrium (LD) measure D' was calculated between the SNPs using the Haploview program, version 3.2 [71], which applies an approach similar to the partition-ligation-expectation-maximization algorithm [72]. Haplotype blocks were constructed using the LD block parameters [73], and the D' confidence interval algorithm in the Haploview program. Tests for haplotype frequency difference between the cases and controls were calculated for the haplotype blocks harboring the SNPs having P of <0.05 for allele association using the Haploview program. The SNP rs10838524 in *CRY2* was included in haplotype analysis even though it was not included in any block but was nearby and associated as a single SNP with winter depression.

## Supporting Information

Table S1 All the detected haplotypes of the CRY2 block.
(0.04 MB DOC)
