# Supplementary material for: CRY2 Is Associated with Depression
Source: PLoS One. 2010 Feb 24;5(2):e9407. doi: 10.1371/journal.pone.0009407 (PMC2827563; doi:10.1371/journal.pone.0009407)
Supplement: Table S1 — All the detected haplotypes of the CRY2 block. (0.04 MB DOC) [file pone.0009407.s001.doc]

**Table S1.** All the detected haplotypes of the *CRY2* block.

| SNPs | | Haplotype | Frequency among controls | Frequency among cases | *P* value for case-control difference |
| --- | --- | --- | --- | --- | --- |
| Swedish sample |  | | | | |
| rs7123390-rs10838527-rs3824872 | | GGA | 0.090 | 0.14 | 0.012 |
|  |  | GAC | 0.52 | 0.44 | 0.032 |
|  |  | AAC | 0.28 | 0.29 | 0.79 |
|  |  | GAA | 0.11 | 0.14 | 0.40 |
| Finnish sample |  | | | | |
| rs7123390-rs10838527-rs3824872 | | GGA | 0.13 | 0.09 | 0.22 |
|  |  | GAC | 0.45 | 0.59 | 0.00010 |
|  |  | AAC | 0.29 | 0.21 | 0.012 |
|  | | GAA | 0.13 | 0.11 | 0.29 |
| HapMap CEU^a^ |  | | | | |
| rs7123390-rs10838527-rs3824872 | | GGA | 0.16 | NA | NA |
|  |  | GAC | 0.47 | NA | NA |
|  |  | AAC | 0.28 | NA | NA |
|  |  | GAA | 0.075 | NA | NA |

^a^Haplotype frequencies for the CEU population (CEPH (Utah residents with ancestry from northern and western Europe)) were downloaded from [www.hapmap.org](http://www.hapmap.org/). NA, not available.
